# Amphetamine Elicits Opposing Actions on Readily Releasable and Reserve Pools for Dopamine

**DOI:** 10.1371/journal.pone.0060763

**Published:** 2013-05-03

**Authors:** Dan P. Covey, Steven A. Juliano, Paul A. Garris

**Affiliations:** School of Biological Sciences, Illinois State University, Normal, Illinois, United States of America; University of Chicago, United States of America

## Abstract

Amphetamine, a highly addictive drug with therapeutic efficacy, exerts paradoxical effects on the fundamental communication modes employed by dopamine neurons in modulating behavior. While amphetamine elevates tonic dopamine signaling by depleting vesicular stores and driving non-exocytotic release through reverse transport, this psychostimulant also activates phasic dopamine signaling by up-regulating vesicular dopamine release. We hypothesized that these seemingly incongruent effects arise from amphetamine depleting the reserve pool and enhancing the readily releasable pool. This novel hypothesis was tested using in vivo voltammetry and stimulus trains of varying duration to access different vesicular stores. We show that amphetamine actions are stimulus dependent in the dorsal striatum. Specifically, amphetamine up-regulated vesicular dopamine release elicited by a short-duration train, which interrogates the readily releasable pool, but depleted release elicited by a long-duration train, which interrogates the reserve pool. These opposing actions of vesicular dopamine release were associated with concurrent increases in tonic and phasic dopamine responses. A link between vesicular depletion and tonic signaling was supported by results obtained for amphetamine in the ventral striatum and cocaine in both striatal sub-regions, which demonstrated augmented vesicular release and phasic signals only. We submit that amphetamine differentially targeting dopamine stores reconciles the paradoxical activation of tonic and phasic dopamine signaling. Overall, these results further highlight the unique and region-distinct cellular mechanisms of amphetamine and may have important implications for its addictive and therapeutic properties.

## Introduction

Amphetamine (AMPH) is both addictive, with several notable episodes of widespread abuse worldwide, and therapeutic, for treating narcolepsy, attention deficit hyperactivity disorder, obesity, and traumatic brain injury [1], [2]. While there is little debate that behavioral effects of this important psychostimulant are associated with a hyperdopamine state [3]–[6], the underlying mechanisms by which this condition manifests have been the subject of intense study. Two, what ostensibly appear to be mutually exclusive, views have emerged. On the one hand, AMPH enhances tonic dopamine signaling by reversing dopamine transporter (DAT) direction, leading to a non-exocytotic, action potential-independent type of release or “efflux” that is driven by vesicular depletion and the redistribution of dopamine to the cytosol [7], [8]. On the other hand, AMPH enhances phasic dopamine signaling by promoting burst firing of dopamine neurons [9], [10], inhibiting dopamine uptake [11], [12], and up-regulating vesicular dopamine release [13], [14]. How AMPH concurrently activates tonic and phasic dopamine signaling, the two fundamental modes of communication used by dopamine neurons [15], yet elicits opposing actions on vesicular dopamine stores is perplexing and unresolved.

Presynaptic neurotransmitter vesicles are functionally and anatomically segregated into at least three distinct pools, readily releasable, recycling, and reserve, that are interrogated by electrical stimulation of short, intermediate, and long duration, respectively [16]. Distinct vesicular stores have also been proposed to contribute to exocytotic dopamine release in a stimulus-dependent manner [17]–[20]. At the cellular level, AMPH exerts differential actions on dopamine vesicle populations [21]–[23]. Moreover, although not systematically evaluated to assess distinct vesicular stores, AMPH effects on electrically evoked levels of extracellular dopamine in the striatum *in vivo* are stimulus-dependent, with increases revealed by short trains and decreases by long trains [24], [25]. It is thus interesting to speculate that AMPH depleting the reserve pool drives tonic dopamine signaling by providing a source of cytosolic dopamine for efflux, but enhancing the readily releasable pool drives phasic dopamine signaling by augmenting vesicular dopamine release.

Here we use *in vivo* voltammetry and vary stimulus duration to test the novel hypothesis that AMPH elicits opposing actions on dopamine stores. In support of this hypothesis, we show in the dorsal striatum that AMPH increased exocytotic dopamine release evoked by a short train, which interrogates the readily releasable pool, but decreased release evoked by a long train, which interrogates the reserve pool. A concurrent augmentation of tonic and phasic dopamine signaling was also observed. Vesicular depletion and enhanced tonic signaling appear to be linked because these effects were specific to AMPH and not cocaine, and to the dorsal but not ventral striatum, whereas activation of vesicular release and phasic signaling generalized across psychostimulants and striatal sub-regions. Our results thus support a model of AMPH differentially targeting vesicular stores to reconcile its paradoxical effects on dopamine neurons and identify regionally distinct actions of this psychostimulant in the striatum that may relate to its addictive and therapeutic properties.

## Methods

### Experimental Design

**Figure 1:**
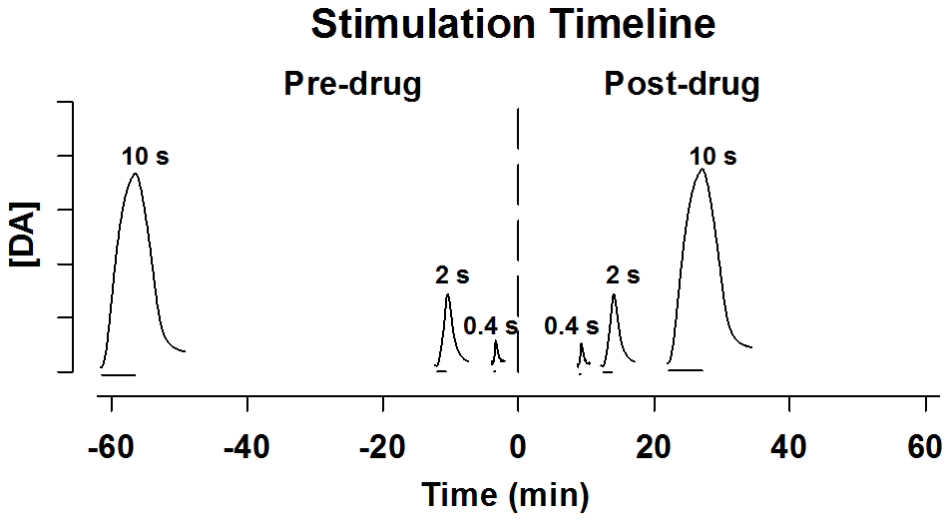
Experimental timeline. Three stimulation trains with different durations (0.4 s, 2 s, and 10 s), indicated by the horizontal line under each evoked response, were applied before and after psychostimulant administration at time 0 min. Note that evoked responses are on a second timescale, while the overall design is shown in minutes.

The experimental design is shown in Figure 1. Three durations of stimulus trains, short (0.4 s), intermediate (2 s), and long (10 s), were applied to each animal and repeated after administration of the saline control or drug treatment. A frequency of 60 Hz was used for all stimulations. Stimulus current was ±300 µA for long and intermediate trains, and ±125 µA for the short train. The lower current intensity was selected for the short train to elicit evoked responses mirroring the amplitude and dynamics of naturally occurring phasic dopamine transients [26]. As such, we refer to these responses as “phasic-like”. This short train is also reinforcing in the operant paradigm of intracranial self-stimulation [27]. Sufficient time was allowed between trains for evoked responses to recover (5 s per pulse; [28]). Extracellular dopamine was measured in urethane-anesthetized rats by fast-scan cyclic voltammetry (FSCV) at a carbon fiber microelectrode (CFM) implanted in the dorsal and ventral striatum, as described previously [12]. Vesicular dopamine release was resolved from dopamine uptake for all evoked responses [28], [29]. A low (1 mg/kg, i.p.) and high (10 mg/kg, i.p.) dose of AMPH was evaluated to assess dose-dependent effects. A high dose of cocaine (40 mg/kg i.p.) was evaluated for comparison.

### Animals

Adult male Sprague-Dawley rats (∼350–400 g), purchased from Harlan (Indianapolis, IN, USA), were housed under standard conditions of lighting and temperature. Food and water were provided *ad libitum*. Protocols were approved by the Institutional Animal Care and Use Committee of Illinois State University. Care was in accordance with NIH guidelines (publication 86–23).

### Surgery

Rats were anesthetized with urethane (1.6 g/kg, i.p.) and immobilized in a stereotaxic frame (David Kopf Instruments, Tujunga, CA, USA). Deltaphase Isothermal Pads (Braintree Scientific, Braintree, MA, USA) maintained core temperature throughout surgery. Burr holes were drilled overlying targeted regions, dura was removed, and electrodes lowered along a vertical trajectory using stereotactic coordinates obtained from a brain atlas based on a flat-skull position [30] and utilizing bregma and dura as reference points. All coordinates, anteroposterior (AP), mediolateral (ML) and dorsoventral (DV) are given in mm. The stimulating electrode targeted the medial forebrain bundle (AP: −4.6, ML: +1.4, DV: −7.0), and a CFM targeted the dorsal (AP: +1.2, ML: +3.0, DV: −4.5 to 5.0) and ventral (AP: +1.2, ML: +2.0, DV: −6.5 to 7.5) striatum. The reference electrode, a chloridized silver wire, was placed in the contralateral superficial cortex.

### Electrochemistry

FSCV was performed by a Universal Electrochemistry Instrument (UEI; Department of Chemistry Electronic Shop, University of North Carolina, Chapel Hill, NC, USA), which was computer controlled by commercially available software (ESA Bioscience, Chelmsford, MA, USA). The potential of the CFM was linearly scanned at 10 Hz from a resting value of −0.4 V to 1.3 V (versus the reference electrode) and back again at a rate of 400 V/s. The peak oxidation current for dopamine recorded during each scan was converted to a concentration based on post-calibration of the CFM using flow-injection analysis in a buffer consisting of 150 mM sodium chloride with 15 mM TRIS and adjusted to a pH of 7.4 [31]. Dopamine was identified from the background subtracted voltammogram [32].

### Electrical Stimulation

Electrical stimulation was computer generated and consisted of biphasic pulses (2 ms each phase). Stimulus trains were applied to a twisted bipolar stimulating electrode (Plastics One, Roanoke, VA, USA) through a constant-current generator and optical isolator (NL 80, Neurolog, Medical Systems, Great Neck, NY, USA).

### Data Analysis

Dopamine responses electrically evoked by short and medium stimulations were analyzed for maximal concentration ([DA]_max_) and parameters described vesicular dopamine release and dopamine uptake according to [28]:
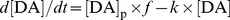
(1)where [DA]_p_ is the concentration of dopamine released per stimulus pulse, f is the frequency of stimulation, and k is the first-order term describing dopamine uptake. Data were best fit to Equation 1 using non-linear regression with a simplex algorithm [29]. First-order, as opposed to Michaelis-Menten, kinetics was selected to characterize dopamine uptake because of concern that AMPH alters both K_m_ and V_max_, which is difficult to resolve with *in vivo* voltammetry [13], [14]. However, similar AMPH-induced changes in [DA]_p_, the focus of the present study, have been reported using both kinetic models [13]. Dopamine responses evoked by long trains were analyzed for vesicular dopamine release using single curve analysis [29]. The reason is that Equation 1 assumes that vesicular dopamine release is constant, and AMPH clearly caused time-dependent changes in recordings evoked by long trains as evident by the pronounced slowing of the upward slope during the train, especially in the dorsal striatum. In single curve analysis, which does not assume a kinetic mechanism for dopamine uptake, the slope of the downward portion of the evoked signal (i.e., uptake) is subtracted from the upward portion (i.e., release - uptake) to calculate vesicular dopamine release:
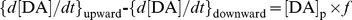
(2)The only assumption of single curve analysis regarding uptake is that rates governing up- and downward portions are identical at the same dopamine concentration, which is also the same assumption as in Equation 1. It should be emphasized that because of DAT reversal, uptake measured in the presence of AMPH more faithfully represents net dopamine clearance, i.e., the difference between extracellular removal by uptake and addition by efflux [12], [33]. Nevertheless, the combination of these effects is accounted for in the analysis, which permits a direct determination of vesicular dopamine release (i.e., [DA]_p_).

Non-electrically evoked changes in extracellular dopamine representing tonic and phasic dopamine signaling were chemically resolved from the FSCV recordings with principal component regression (PCR) using dopamine, pH and background drift as analytes [34], [35]. For training sets, dopamine and pH changes were obtained from the electrically evoked responses, whereas background drift was obtained during baseline recording in the time between stimulations. PCR was performed sequentially on 5-min epochs. Spontaneously occurring dopamine transients were identified and characterized with peak-finding software (Mini-Analysis, Synaptosoft, Decatur, GA, USA).

### Statistical Analysis

When appropriate, data are presented as the mean ± SEM. [DA]_max_ and [DA]_p_ were statistically analyzed using a two-way ANOVA with drug treatment and stimulus duration as independent variables, followed by sequential Bonferroni *post hoc* tests. Effects of drug treatment on k were analyzed using a one-way ANOVA with a Tukey's *post hoc* test. Tonic dopamine levels were statistically analyzed using a one-way ANOVA with repeated measures. Statistical analysis was performed using SPSS Version 18 for Windows (SPSS). Significance was set at *p*<0.05.

### Drugs

Urethane, cocaine hydrochloride, and d-amphetamine sulfate were purchased from Sigma (St. Louis, MO, USA). All drugs were dissolved in 150 mM NaCl prior to injection. *d*-amphetamine and cocaine doses were determined by base weight.

## Results

### Psychostimulant effects on evoked dopamine levels

**Figure 2:**
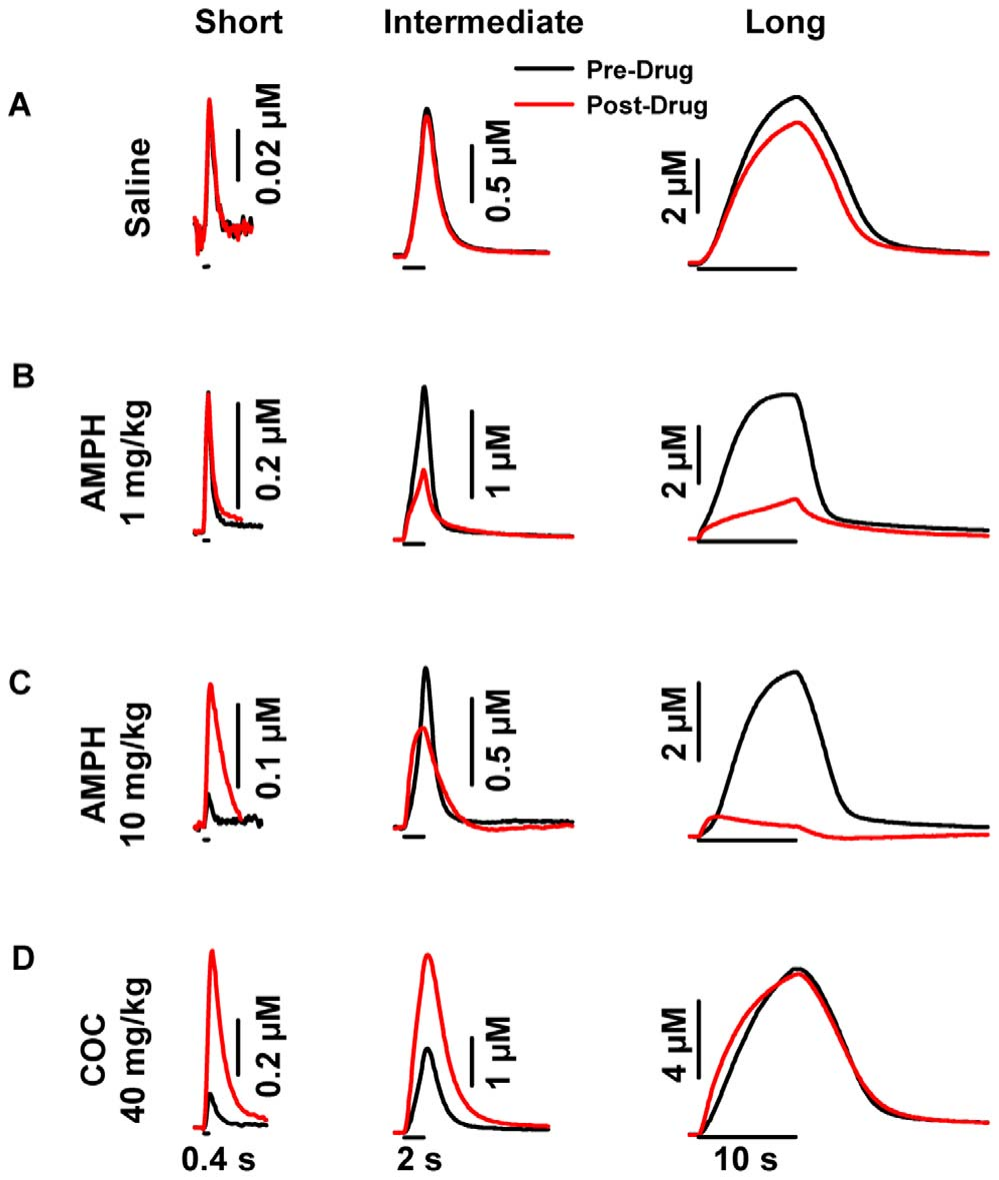
Representative psychostimulant- and stimulation-dependent effects on evoked dopamine dynamics in the dorsal striatum. **A.** Saline. **B.** 1 mg/kg AMPH. **C.** 10 mg/kg AMPH. **D.** 40 mg/kg cocaine (COC). AMPH and cocaine altered the amplitude of evoked dopamine signals in the dorsal striatum, while saline had no effect. In contrast to cocaine, there was an inverse relationship between stimulus duration and evoked dopamine amplitude following AMPH. Application of the stimulus train is indicated by the solid line underneath each representative response for short (left), intermediate (middle) and long (right) durations.

**Figure 3:**
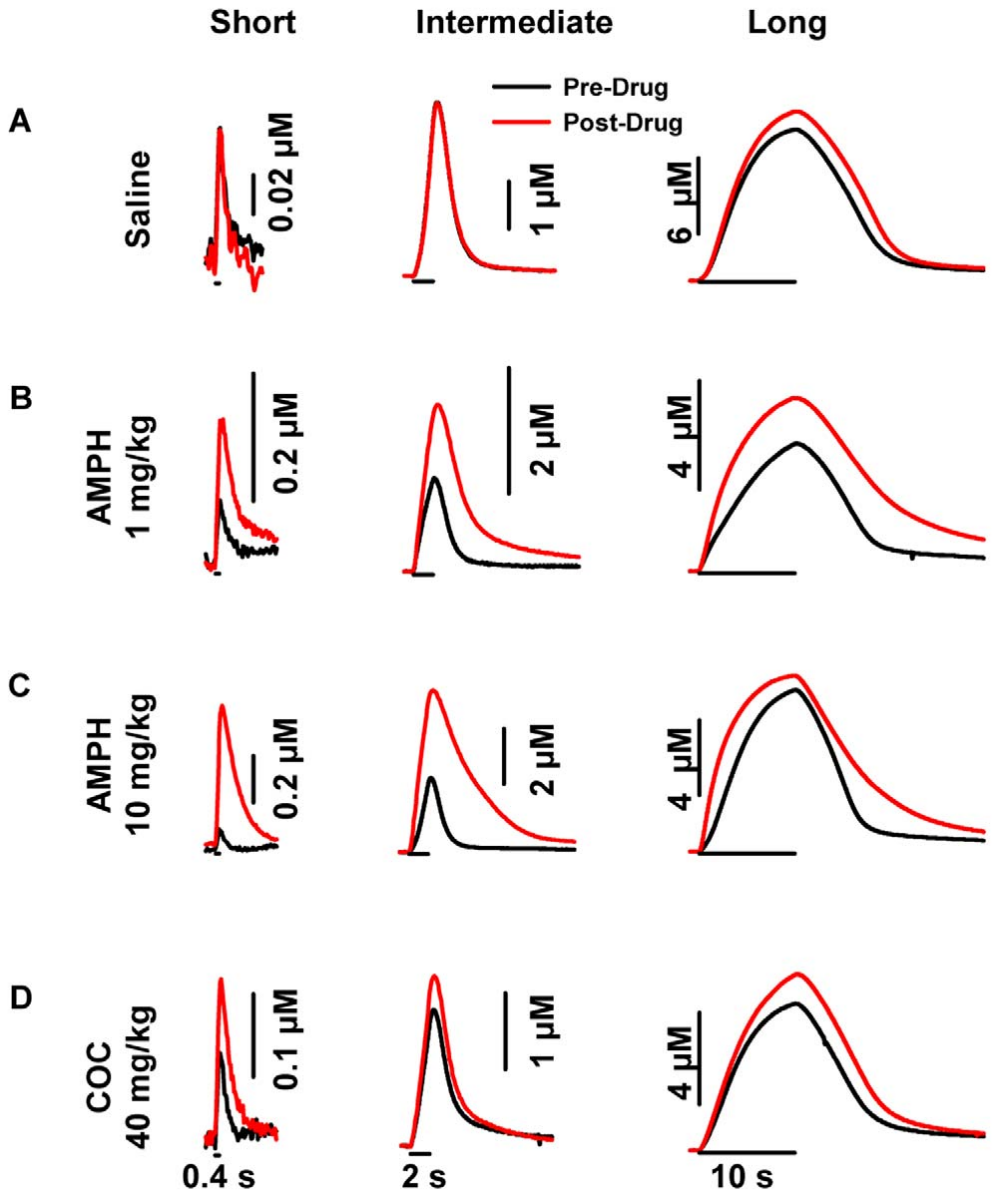
Representative psychostimulant- and stimulation-dependent effects on evoked dopamine dynamics in the ventral striatum. **A.** Saline. **B.** 1 mg/kg AMPH. **C.** 10 mg/kg AMPH. **D.** 40 mg/kg cocaine (COC). AMPH and cocaine increased evoked dopamine amplitude for at stimulus durations in the ventral striatum, while saline had no effect. Application of the stimulus train is indicated by the solid line underneath each representative response for short (left), intermediate (middle) and long (right) durations.

**Figure 4:**
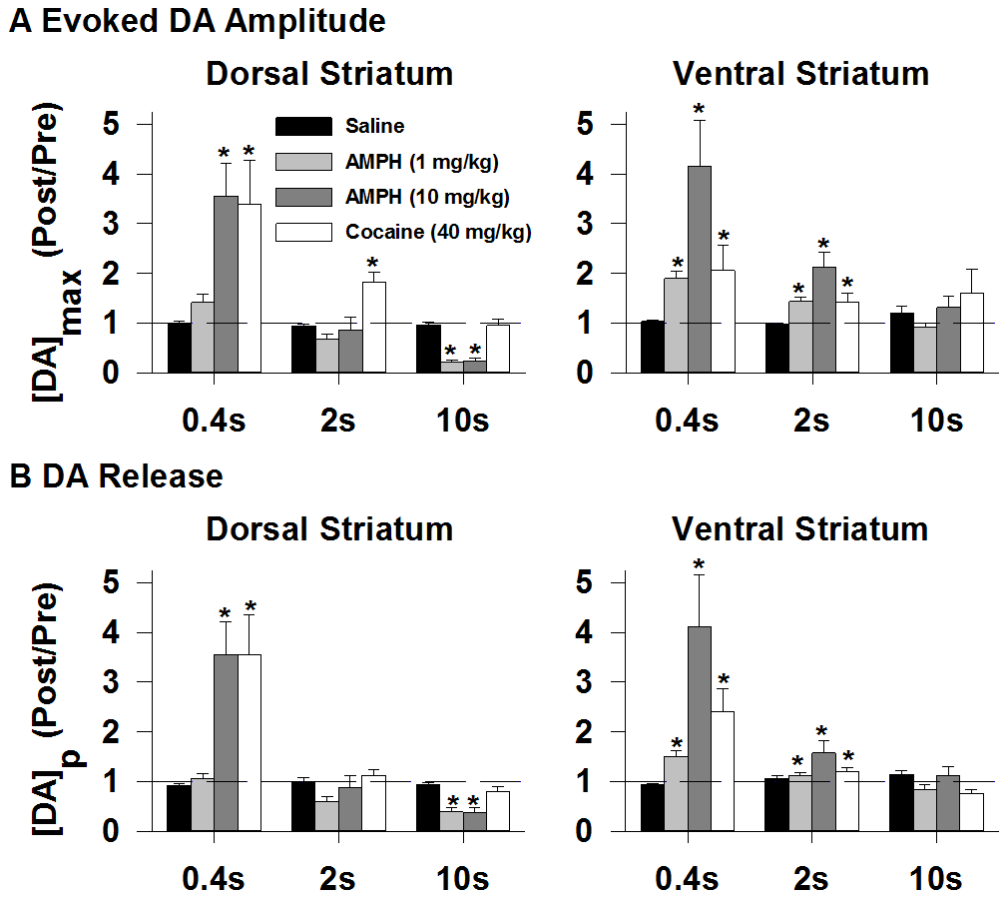
Averaged psychostimulant- and stimulation-dependent effects. **A.** The maximal concentration of electrically evoked dopamine ([DA]_max_). **B.** Vesicular release ([DA]_p_). Stimulus duration is shown along the x axis. Psychostimulants differentially elicited stimulus-dependent effects on [DA]_max_ and [DA]_p_. Data are the ratio of post-drug over pre-drug response (Post/Pre) for the dorsal (left) and ventral (right) striatum and are expressed as mean ± SEM. *, significantly different from saline (*p*<0.05).

Individual recordings of electrically evoked dopamine levels collected during the four treatments are shown in Figure 2 for the dorsal striatum and Figure 3 for the ventral striatum. Average results for [DA]_max_, the maximal concentration of the evoked signal, and obtained from these recordings are shown in Figure 4A (left, dorsal striatum; right, ventral striatum). Both individual responses and averaged results demonstrate drug-, dose-, stimulus-, and region-dependent effects, and four general observations can be made. First, psychostimulant effects were inversely related to stimulus duration in both striatal regions. Second, AMPH but not cocaine decreased [DA]_max_ evoked by the long train, and this only occurred in the dorsal striatum. Third, AMPH was more proficient in increasing [DA]_max_ evoked by the short train in the ventral striatum, whereas cocaine elicited greater effects in the dorsal striatum. And fourth, the high dose of AMPH was more proficient at increasing [DA]_max_ during short trains in both striatal regions compared to the low dose. Statistical analysis of [DA]_max_ revealed a significant effect of drug treatment in the dorsal (F_3,75_ = 13.45, *p* = <0.001) and ventral (F_3,74_ = 8.81, *p*<0.001) striatum, a significant effect of stimulus duration in the dorsal (F_2,75_ = 47.94, *p*<0.001) and ventral (F_2,74_ = 13.96, *p*<0.001) striatum, and a significant interaction in the dorsal (F_6,75_ = 8.45, *p*<0.001) and ventral (F_6,74_ = 3.08, *p*<0.01) striatum. In the dorsal striatum, 10 mg/kg AMPH and 40 mg/kg cocaine significantly (*p*<0.002) increased [DA]_max_ evoked by the short train, but only cocaine was effective at the intermediate train (*p*<0.001). Both doses of AMPH (1 and 10 mg/kg) significantly (*p*<0.001) decreased [DA]_max_ evoked by the long train, whereas cocaine was without effect. In the ventral striatum, both doses of AMPH and cocaine significantly (*p*<0.01) increased [DA]_max_ evoked by short and intermediate trains, but were without effect with the long train.

### Psychostimulant effects on vesicular dopamine release and dopamine uptake

Observed psychostimulant-induced changes in [DA]_max_ could arise from altered vesicular dopamine release and/or dopamine uptake, because both mechanisms regulating extracellular dopamine in the striatum operate concurrently during the stimulus train [28]. Evoked responses were therefore analyzed to determine the respective contributions of these presynaptic mechanisms to [DA]_max_. Figure 4B shows vesicular dopamine release ([DA]_p_). Overall, [DA]_p_ and [DA]_max_ (Fig. 4A) tracked each other well. Statistical analysis of [DA]_p_ revealed a significant effect of drug treatment in the dorsal (F_3,73_ = 8.36, *p*<0.001) and ventral (F_3,72_ = 6.79, *p*<0.001) striatum, a significant effect of train duration in the dorsal (F_2,73_ = 30.45, *p*<0.001) and ventral (F_2,72_ = 19.53, *p*<0.001) striatum, and a significant interaction in the dorsal (F_6,73_ = 6.33, *p*<0.001) and ventral (F_6,72_ = 4.26, *p*<0.001) striatum. In the dorsal striatum, 10 mg/kg AMPH and cocaine significantly increased [DA]_p_ for the short train (*p*<0.02). 1 mg/kg AMPH was without effect, and no treatment had significant effects for the intermediate train. Both doses of AMPH significantly decreased [DA]_p_ for the long train (*p*<0.01), while cocaine had no effect. All drug treatments significantly increased [DA]_p_ in the ventral striatum for both short and intermediate trains (*p*<0.03) but were without effect for the long stimulation.

Psychostimulant effects on dopamine uptake are shown in Table 1. Low- and high-dose AMPH and cocaine robustly decreased dopamine uptake (k) to a similar degree in both striatal regions. Statistical analysis of k revealed a significant effect of drug treatment in both the dorsal (F_3,54_ = 10.53, *p*<0.001) and ventral (F_3,54_ = 15.80, *p*<0.001) striatum. Each drug treatment significantly decreased dopamine uptake compared to saline control in both striatal regions (*p*<0.01). AMPH- and cocaine-mediated uptake inhibition is consistent with our previous work using Michaelis-Menten kinetics [12], [29], [31], and the degree of inhibition was similar to our previous work using first-order kinetics [14], as is used here. This result, indicating no distinct effects of drug treatment or striatal region on dopamine uptake, and the excellent correspondence between [DA]_max_ and [DA]_p_ shown in Figure 3, suggest that psychostimulant-induced changes in [DA]_max_ evoked by the trains used in this study are dominated by changes in vesicular dopamine release. The one overt exception is the intermediate train in the dorsal striatum, where cocaine increased [DA]_max_ without a corresponding change in [DA]_p_. In this case, reduced dopamine uptake dominates the increase in [DA]_max_. Overall, these results demonstrate that AMPH and cocaine increase vesicular dopamine release in both striatal regions with the short train but that AMPH decreases vesicular dopamine release in the dorsal striatum with the long train.

**Table 1:** Psychostimulant effects on dopamine uptake.

	Saline	AMPH (1 mg/kg)	AMPH (10 mg/kg)	Cocaine (40 mg/kg)
**Dorsal**	0.96±0.05	0.57±0.04**	0.53±0.08**	0.66±0.06**
**Ventral**	0.95±0.04	0.62±0.05**	0.50±0.08**	0.60±0.05**

Data are the mean ± SEM.

** , significantly different from saline (*p*<0.01).

### Psychostimulant effects on tonic dopamine signaling

**Figure 5:**
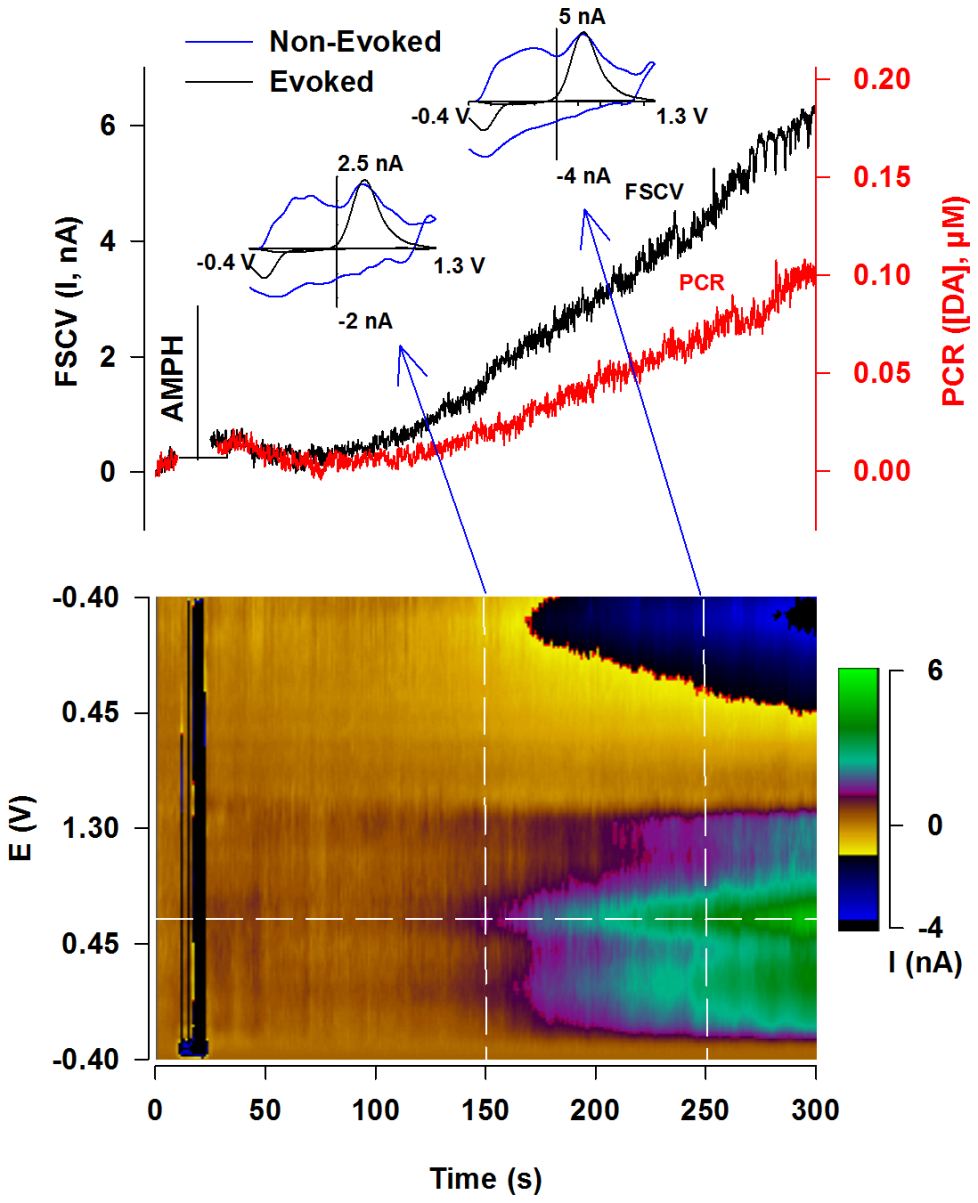
Representative effects of AMPH on tonic dopamine levels in the dorsal striatum. The black line (left y axis) in the top panel shows background-subtracted current, and pseudocolor plot underneath displays all background-subtracted cyclic voltammograms immediately following administration of the high dose (10 mg/kg) of AMPH. Current, which was measured at the peak oxidative potential for dopamine (horizontal white line on the pseudocolor plot), was converted to dopamine concentration (red line, right y axis) using PCR. **INSET.** Background-subtracted cyclic voltammograms taken at 150 s and 250 s (blue arrows, blue line) and from the post-drug electrically evoked (60 Hz, 0.4 s) dopamine signal (black line).

Figure 5 shows a representative background-subtracted FSCV recording (black) collected immediately surrounding the time of injecting high-dose AMPH. This non-electrically evoked trace, representing current measured at the peak oxidative potential for dopamine (i.e., along the horizontal white line of the pseudocolor plot below), gradually increases across the 5-min epoch. Individual voltammograms collected along the two vertical white lines of the pseudocolor plot (blue) are overlaid with a dopamine voltammogram collected during electrical stimulation (black) earlier in this recording (data not shown). While there is evidence for dopamine in the individual voltammograms and in the sequential voltammograms displayed in the pseudocolor plot for this non-electrically evoked trace, other analytes obscure its selective measurement with FSCV alone. However, PCR (red) resolves the dopamine component of this FSCV recording, demonstrating an activation of tonic dopamine signaling by AMPH.

**Figure 6:**
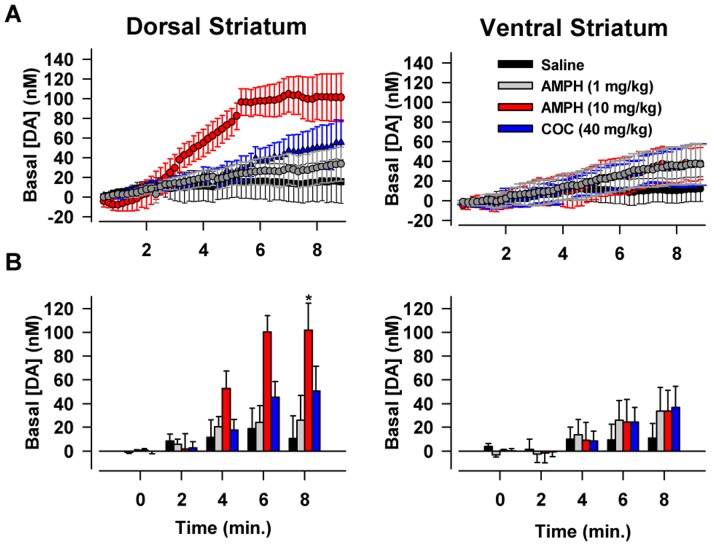
Averaged psychostimulant-induced increases in tonic dopamine levels. **A.** Time course of the effects of AMPH and cocaine (COC) on tonic dopamine levels. Dopamine concentrations were determined using PCR and averaged across 10-s bins. The time period is the epoch immediately following drug injection and prior to the first post-drug. **B.** Dopamine levels from **A.** above but only shown at two-minute intervals. These data were used for statistical analysis. Data in the dorsal (left) and ventral (right) striatum are expressed as mean ± SEM. *, significantly different from other treatments (*p*<0.05).

Figure 6 shows the average effects of the four treatments on tonic dopamine signaling as determined by PCR analysis for the first 10 min of the FSCV recording after drug injection, which is just prior to the first stimulation of the post-drug period (see Fig. 1). This initial recording period was selected for analysis to avoid interactions between stimulation, psychostimulants, and tonic dopamine signaling. In the dorsal striatum (Fig. 6A), AMPH (10 mg/kg) elicited the fastest and largest increase in tonic dopamine levels. Statistical analysis revealed a significant effect of treatment (F_3,22_ = 3.38, *p* = 0.04), time (F_4,22_ = 11.99, *p*<0.001), and interaction (F_12,22_ = 2.13, *p* = 0.03). A *post hoc* comparison of the average change across the last two minutes of the time course (INSET) revealed that only 10 mg/kg AMPH significantly increased tonic dopamine levels compared to saline (*p*<0.01). In the ventral striatum (Fig. 6B) region, the effects of each psychostimulant were largely indistinguishable from each other and only slightly different than the saline control. Statistical analysis revealed a significant effect of only time (F_4,22_ = 3.90, *p* = 0.02). Overall, these results suggested that AMPH is more effective at increasing tonic dopamine signaling than cocaine and in the dorsal compared to the ventral striatum initially after drug injection.

### Psychostimulant effects on phasic dopamine signaling

**Figure 7:**
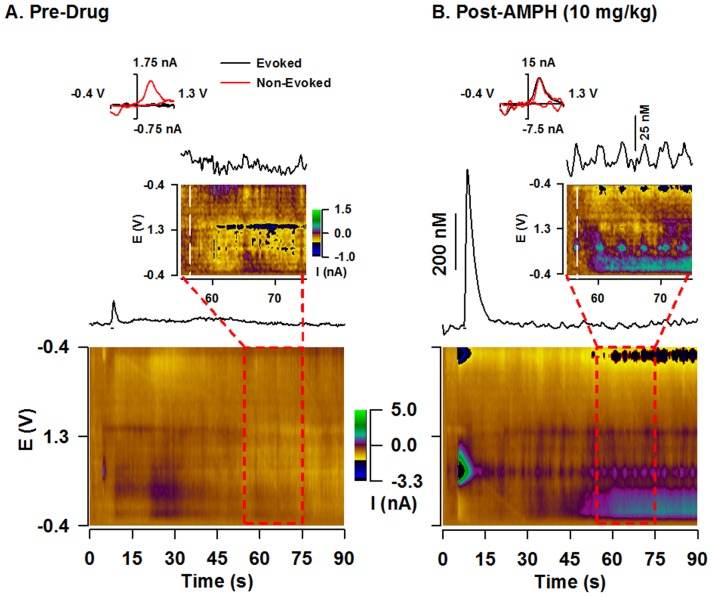
Representative effects of AMPH on phasic dopamine signaling in the ventral striatum. **A.** Pre-drug. **B.** Post-AMPH. Traces show 90 s of a recording with a short-duration (0.4 s) stimulation applied at 5 s (see line underneath). The color plot serially displaying all background-subtracted cyclic voltammograms is shown underneath. **INSET.** Time-expanded view. Individual background-subtracted voltammograms are shown at the top left and compare dopamine collected during the evoked phasic-like response (black line) to pre-drug baseline (**A.**) or a dopamine transient collected post-drug (**B.**) as indicated by vertical white line in the pseudocolot plot (red line).

Increased [DA]_max_ of phasic-like dopamine responses evoked by the short train (Figs. 2, 3, 4) suggests that both amphetamine and cocaine activate phasic dopamine signaling. These results are thus consistent with the two psychostimulants augmenting naturally occurring dopamine transients in awake, freely behaving animals [13], [36], [37]. While psychostimulant-induced burst firing of dopamine neurons is typically blunted under anesthesia [38] unless revealed by D2 antagonists [9], [10], dopamine transients are elicited by AMPH in a subset of animals in this preparation [14]. An example of this activation is shown in Figure 7. Before drug injection, the dopamine response evoked by the short train was small and no dopamine transients were observed (Fig. 7A). In sharp contrast, high-dose AMPH dramatically increased this evoked phasic-like signal, mediated by augmented vesicular dopamine release and inhibited dopamine uptake (Fig. 4 and Table 1), and transient frequency (Fig. 7B). To better view the presence or absence of dopamine transients, FSCV recordings are expanded in the INSET. These short-lived, non-electrically evoked deflections were identified as dopamine by the sequential voltammograms displayed in the pseudocolor plot below each trace and by the overlay of the individual voltammogram for the transients (black) with that obtained from the evoked signal established to be dopamine (red) to the left in the INSET.

**Figure 8:**
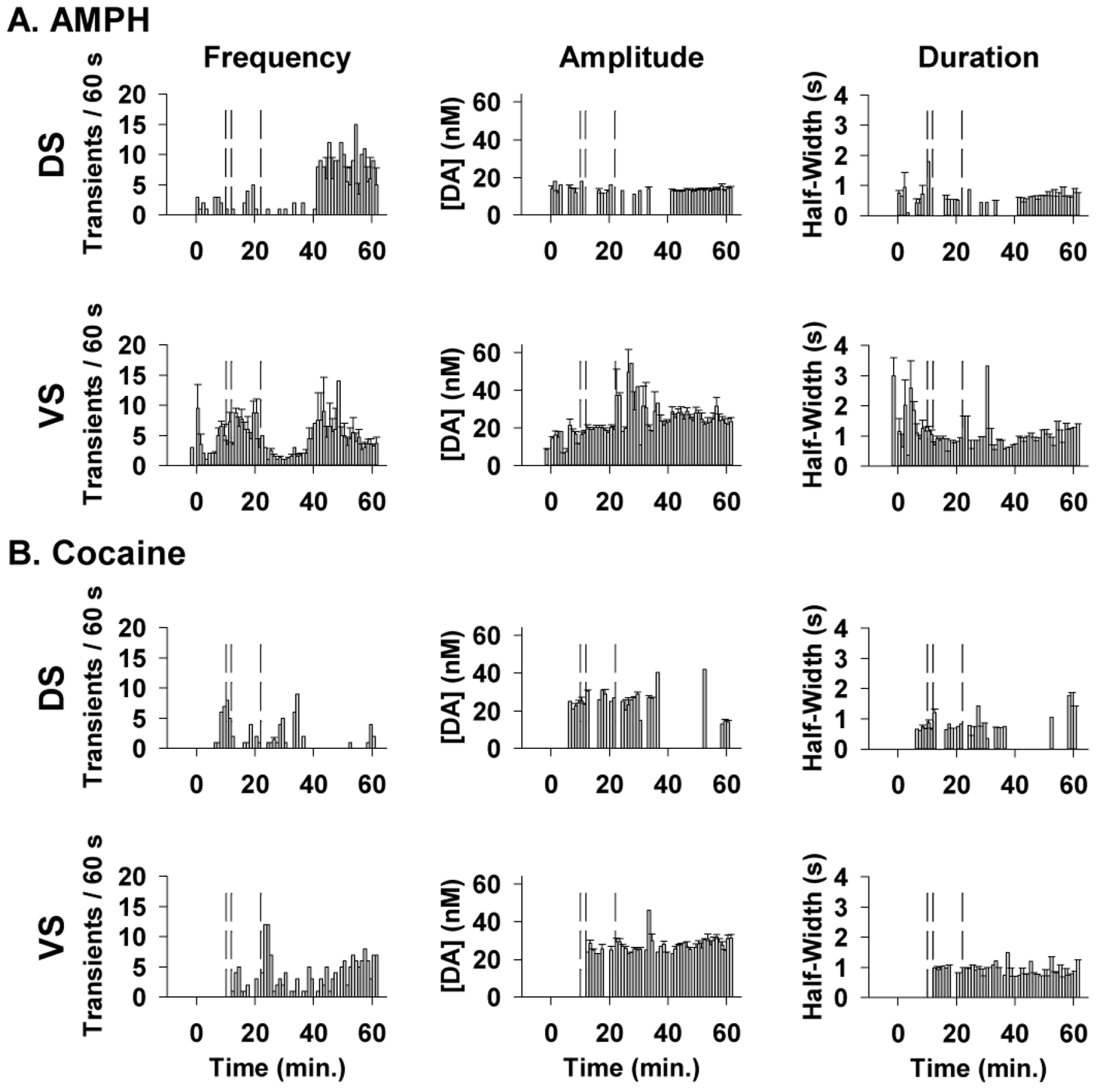
Averaged effects of psychostimulants on dopamine transients. **A.** AMPH. **B.** Cocaine. Dopamine transients were analyzed in terms of frequency (left), amplitude (middle) and duration (right) in both the dorsal and ventral striatum (DS and VS, respectively). Data are transient characteristics compiled into 60-s bins and express as the mean ± SEM. Each histogram shows transient characteristics for the 10 minutes before, and the 65 minutes after drug injection (at time 0 min). Phasic dopamine transients were observed following AMPH in 3 of 7 animals in the dorsal striatum and 5 of 7 in the ventral striatum and following cocaine in 3 of 7 animals in the dorsal striatum and 1 of 7 animals in the ventral striatum.

To complement evoked phasic-like responses, we thus analyzed these dopamine transients to obtain a more physiological assessment of psychostimulant effects on phasic dopamine signaling. Figure 8 shows the time course of dopamine transients for high-dose AMPH (Panel A) and cocaine (Panel B) in the dorsal and ventral striatum (top and bottom, respectively) in the subset of animals where this phasic activity was observed (see legend for details). Transients were analyzed for frequency (left), amplitude (middle), and duration (right). Time 0 min is drug injection. The time when short, intermediate and long trains were applied during the post-drug period is demarcated by vertical dashed lines at 10, 12 and 22 min, respectively. High-dose AMPH and cocaine activated dopamine transients in both striatal subregions. Transients were rarely observed during pre-drug recording and were not observed after saline or low-dose AMPH. Both psychostimulants increased the frequency of dopamine transients to a greater extent in the ventral compared to the dorsal striatum, and AMPH was more effective than cocaine in both striatal subregions. The onset of dopamine transient activation was also slower for cocaine. A clear inhibition and rebound in transient frequency was observed following the long train in both the dorsal and ventral striatum after AMPH. This effect is most likely related to feedback inhibition by released dopamine [39], with the additional combination of AMPH and the long train depleting vesicular dopamine release in the dorsal striatum (Fig. 4). Overall, results for dopamine transients are consistent with those for evoked phasic-like responses and suggest that AMPH and cocaine activate phasic dopamine signaling.

## Discussion

The goal of the present study was to reconcile the paradoxical effects of AMPH on dopamine neurons. To this end, we tested the novel hypothesis that AMPH depletes the reserve pool but up-regulates the readily releasable pool. This hypothesis was formulated based on three key observations reported in the literature. First, dopamine neurons contain distinct vesicular storage pools. Second, different train durations interrogate different vesicular storage pools. And third, AMPH effects on electrically evoked dopamine levels in the dorsal striatum appear inversely related to train duration. We tested this hypothesis using a novel experimental design. When taken together, our results support a model of AMPH activating tonic dopamine signaling by depleting the reserve pool to drive non-exocytotic efflux, but activating phasic dopamine signaling by up-regulating the readily releasable pool to drive vesicular dopamine release.

### Experimental Design

Four features highlight the utility of the experimental design. First, different train durations, selected to demonstrate stimulus-dependent AMPH effects, were applied to the same animal. Although this strategy fosters inter-animal comparisons, it also risks train interactions because dopamine release depends upon stimulation history [40]. However, stability of the saline control and replicating stimulus-dependent AMPH effects demonstrated previously in separate animals indicated that judicial spacing of trains was sufficient to minimize interaction. Second, evoked dopamine dynamics were resolved into the respective contributions of vesicular release and uptake. Most previous studies examining stimulus-dependent AMPH effects report dopamine levels only and therefore do not directly assess release. Third, the status of dopamine storage pools was related to tonic and phasic dopamine signaling. Such an integrated view of AMPH action has not been available. And fourth, we compared AMPH to cocaine, which is recognized to inhibit DAT and increase vesicular release, but not to deplete vesicular stores *in vivo*.

### AMPH enhances tonic and phasic dopamine signaling

Tonic dopamine signaling, which is characterized by a steady-state basal level of dopamine and controlled by slow irregular firing of dopamine neurons and presynaptic input [41], enables movement, cognition and motivation [15]. AMPH robustly increases tonic dopamine levels measured by microdialysis [6], but comparatively greater elevations in dialysate dopamine relative to other DAT-inhibiting psychostimulants such as cocaine [5] are attributed to the unique action of AMPH eliciting non-exocytotic efflux [7], [8]. We show here that only high-dose AMPH increased tonic dopamine levels and this only occurred in the dorsal striatum. Analytical differences between measurement techniques may have contributed to discrepancies between the present measures with FSCV and microdialysis studies [42]. While FSCV excels at fast measurements with a small probe, inherent limitations in selectivity require the use of statistical methods such as PCR to resolve the dopamine component of tonic changes [34]. Microdialysis exhibits superior selectivity but suffers from implantation damage due to the considerably larger probe that overestimates the increase in tonic dopamine levels with dopamine uptake inhibitors [42]. Thus, measurements of tonic dopamine levels using both approaches should be carefully scrutinized. We should also emphasize that a conservative approach with FSCV was used to minimize error, by only characterizing the first 10-min post-drug epoch and by incorporating background drift as a PCR component [35]. Increases in tonic dopamine levels may thus have occurred after this time. Another consideration when comparing the present and microdialysis studies is anesthesia, which inhibits dopamine neuron firing [43]. However, observed effects of saline and low- and high-dose AMPH on tonic dopamine levels are consistent with un-anesthetized recordings [13]. The contribution of efflux to AMPH-induced increases in tonic dopamine levels measured by FSCV and observed here and elsewhere [13], [14] has not been determined. However, efflux is implicated using the present experimental design because increased tonic levels are associated exclusively with vesicular depletion.

Phasic dopamine signaling, in which burst firing of dopamine neurons generates sub-second changes in extracellular dopamine called transients [44], is important for goal-directed behavior and reinforcement learning [15]. Cocaine activates burst firing [38], the amplitude, frequency and duration of naturally occurring dopamine transients [36], [37], and evoked phasic-like dopamine responses [14], [45]. AMPH has also been shown to augment evoked phasic-like dopamine responses, as well as spontaneously occurring and cue-evoked dopamine transients [13], [14]. Consistent with these previous studies, we show here that both AMPH and cocaine activated evoked phasic-like dopamine responses and dopamine transients. Anesthesia likely attenuated these effects by inhibiting burst firing [43] and phasic activation by psychostimulants [13], [14], [37], [38], [46]. However, awake, freely behaving animals do not tolerate intermediate and long stimulus trains, so anesthesia is required to assess recycling and reserve pools.

### Stimulus-dependent effects of AMPH on [DA]_max_

The present results, obtained by applying different train durations to the same animal, are consistent with previous work applying these same trains individually in separate animals. For example, in the presence of AMPH and in the dorsal striatum, the long train decreased [DA]_max_ [25], [47], [48], the intermediate train elicited minimal to no effect [11], [12], and the short train increased [DA]_max_ [13], [14]. Similar results were obtained in the ventral striatum, except that the long train did not decrease [DA]_max_, which is also consistent with previous work [48]. We additionally extend these studies by comparing AMPH effects to cocaine, which only elicited increases or no change in [DA]_max_, and by determining the underlying change in vesicular dopamine release, which permits analysis of storage pools. Indeed, because both AMPH and cocaine robustly inhibit dopamine uptake (Table 1, [12]–[14], [31], [33], [49]), observed alterations in [DA]_max_ have a complex origin.

### AMPH elicits opposing actions on readily releasable and reserve pools for dopamine

Work with model synapses indicates that readily releasable, recycling, and reserve pools of neurotransmitters are interrogated by short, intermediate, and long duration trains, respectively [16]. We used this approach to investigate the effects of AMPH on dopamine stores. In the dorsal striatum, each stimulus train elicited a distinct action on vesicular dopamine release in the presence of high-dose AMPH: increase, no change, and decrease for short, intermediate, and long trains, respectively. Taken together, these results suggest that AMPH augments the readily releasable pool, exerts no effect on the recycling pool, and depletes the reserve pool in the dorsal striatum. By contrast, the readily releasable pool and to a lesser extent the recycling pool were up-regulated without depletion of the reserve pool by AMPH in the ventral striatum and cocaine in both striatal sub-regions. As a psychostimulant with multiple actions, AMPH could augment vesicular dopamine release by several mechanisms, such as: (1) inhibiting monoamine oxidase [50] and activating tyrosine hydroxylase [51], leading to greater cytosolic dopamine levels, vesicular packaging, and ultimately quantal size; (2) increasing membrane excitability as a DAT substrate [52]; and (3) enhancing exocytosis by liberating vesicular Ca^2+^ stores [53]. Depleting the reserve pool suggests another mechanism, re-distributed cytosolic dopamine being re-packaged by the readily releasable pool. This latter postulate is supported by the greater capacity of this vesicle population to sequester cytosolic dopamine [54], [55]. Moreover, robust depletion of vesicular dopamine stores by AMPH, well established using reduced preparations [21], [22], [53], [56]–[61], appears to occur independently in separate classes of dopamine vesicles [21], [22]. Depletion involves AMPH acting as a weak base to destabilize the proton gradient across vesicles and as a substrate of the vesicular monoamine transporter to inhibit and/or reverse its action [7], [8]. How these mechanisms might differ across dopamine storage pools, as our results would suggest, remains to be determined.

We also do not know why AMPH depleted vesicular dopamine stores in the dorsal but not ventral striatum. One possible origin is regional differences in DAT. For example, DAT binding and V_max_ for dopamine uptake are higher in the dorsal striatum [62], [63], and DAT is more glycosylated with a higher molecular weight in the ventral striatum [64]. Although K_m_ for dopamine uptake is similar in the two regions [31], [63], AMPH is a more potent competitive inhibitor of dopamine uptake in the dorsal compared to the ventral straitum [12]. We are not aware of comparable regional differences in the vesicular monoamine transporter. Another possible origin is regional differences in vesicular dopamine stores. As mentioned above, different classes of dopamine vesicles exhibit different sensitivities to the depleting actions of AMPH [21], [22]. Consistent with region-specific actions of AMPH on vesicular dopamine stores, we have recently shown that AMPH may up-regulate vesicular dopamine release in the ventral striatum by mobilizing the reserve pool but by activating dopamine synthesis and inhibiting dopamine degradation in the dorsal striatum [65]. Different distributions of small, clear and large, dense-core vesicles in the two striatal sub-regions [66] may also contribute to the differential response to AMPH. Clearly, more work needs to be done to resolve the differential depleting effects of AMPH on dopamine vesicles in the dorsal and ventral striatum.

### New model of amphetamine action

We propose a new model of AMPH action: activating tonic dopamine signaling by depleting the reserve pool, which elevates cytosolic dopamine and drives reverse transport through DAT, while concurrently activating phasic dopamine signaling by up-regulating the readily releasable pool, which drives vesicular dopamine release. This model is supported here by the first report of a selective coupling between tonic activation and vesicular depletion coincident with phasic activation and up-regulated vesicular release. Revealing this unique combination of AMPH effects underscores the utility of the experimental design employed. Indeed, slice voltammetry has demonstrated a parallel between robust vesicular depletion and micromolar dopamine efflux, but no measures of phasic signaling or its release component were examined [58], [60], [61]. Moreover, *in vivo* voltammetry has demonstrated concurrent activation of tonic and phasic dopamine signaling and up-regulated vesicular release, but effects on the reserve pool were not assessed [13], [14]. Further supporting our proposed model is that, in contrast to AMPH in the dorsal striatum, AMPH in the ventral striatum and cocaine in both striatal sub-regions did not deplete vesicular stores or elevate tonic dopamine levels, despite phasic activation and up-regulated vesicular release.

Two confounds need addressing. First, coupling between tonic activation and vesicular depletion was not observed for low-dose AMPH in the dorsal striatum. It could be that, while cytosolic dopamine increased as a result of vesicular depletion, low-dose AMPH was insufficient to inhibit monoamine oxidase and prevent its intracellular degradation and/or to reverse DAT direction and cause efflux. Both AMPH effects are dose-dependent [50], [67]. Also consistent with this interpretation is that vesicular depletion alone does not elicit efflux [58] and that both vesicular depletion and blockade of monoamine oxidase are required for cytosolic levels to increase [59]. In contrast, there are other reports demonstrating that increases in cytosolic dopamine alone are sufficient to induce efflux [44], [68]. Second, low-dose AMPH also did not activate phasic dopamine signaling or vesicular dopamine release in the dorsal striatum. However, this lack of response is an anesthesia artifact, because both are enhanced in awake, freely behaving animals [13].

### Implications for psychostimulant neurobiology

We demonstrate fundamentally similar and distinct mechanisms for two major classes of psychostimulants, AMPH representing the so-called dopamine “releasers” (i.e., eliciting non-exocytotic efflux) and cocaine representing the DAT “inhibitors” [33]. While AMPH and cocaine share phasic activation through augmented vesicular dopamine release (Fig. 4, [13], [14], [45], [49], [69]–[71]) and enhanced burst firing [9], [10], [38], they differ in tonic activation. In particular, cocaine requires action potential-dependent mechanisms whereas AMPH does not [72]–[74]. Inhibition of dopamine uptake (Table 1, [12]–[14], [31], [33], [49]) would contribute to augmented tonic and phasic signaling by both psychostimulants. However, activation of vesicular dopamine release may be more important than uptake inhibition, especially for phasic signaling, because release better tracks [DA]_max_ (Fig. 4, [13]).

The neurobiological implications of these psychostimulant actions are not presently known, but they could be profound. Several drugs of abuse have now been demonstrated to augment dopamine transients, including amphetamine, cocaine, nicotine and ethanol [13], [37], [46], [75], [76]. The greater activation of phasic dopamine signaling by abused drugs compared to natural rewards and the subsequent usurpation of normal reward processing to promote addiction [77] may thus represent a unifying mechanism. While both classes of psychostimulants would promote reinforcement learning by activating the direct (“Go”) pathway in the basal ganglia via enhanced phasic signaling and D1 receptor binding, AMPH would more robustly inhibit the indirect (“No Go”) pathway (i.e., disinhibition of behavior) via enhanced tonic signaling and D2 receptor binding [78], because of the added contribution of non-exocytotic efflux. Future directions should also investigate how intrastriatal differences in AMPH action relate to the diverse roles of dopamine signaling in this region for promoting drug reinforcement and addiction [79], [80].

## References

[1] Bales JW, Wagner AK, Kline AE, Dixon CE (2009) Persistent cognitive dysfunction after traumatic brain injury: A dopamine hypothesis. Neurosci Biobehav Rev 33: 981–1003 S0149-7634(09)00045-1 [pii];10.1016/j.neubiorev.2009.03.011 [doi]. 19580914 10.1016/j.neubiorev.2009.03.011 PMC2806224

[2] Howell LL, Kimmel HL (2008) Monoamine transporters and psychostimulant addiction. Biochem Pharmacol 75: 196–217 S0006-2952(07)00538-2 [pii];10.1016/j.bcp.2007.08.003 [doi]. 17825265 10.1016/j.bcp.2007.08.003

[3] Carboni E, Imperato A, Perezzani L, Di Chiara G (1989) Amphetamine, cocaine, phencyclidine and nomifensine increase extracellular dopamine concentrations preferentially in the nucleus accumbens of freely moving rats. Neuroscience 28: 653–661. 2710338 10.1016/0306-4522(89)90012-2

[4] Di Chiara G, Imperato A (1988) Drugs abused by humans preferentially increase synaptic dopamine concentrations in the mesolimbic system of freely moving rats. Proc Natl Acad Sci U S A 85: 5274–5278. 2899326 10.1073/pnas.85.14.5274 PMC281732

[5] Kuczenski R, Segal DS, Aizenstein ML (1991) Amphetamine, cocaine, and fencamfamine: relationship between locomotor and stereotypy response profiles and caudate and accumbens dopamine dynamics. J Neurosci 11: 2703–2712. 1715389 10.1523/JNEUROSCI.11-09-02703.1991 PMC6575248

[6] Kuczenski R, Melega WP, Cho AK, Segal DS (1997) Extracellular dopamine and amphetamine after systemic amphetamine administration: comparison to the behavioral response. J Pharmacol Exp Ther 282: 591–596. 9262319

[7] Fleckenstein AE, Volz TJ, Riddle EL, Gibb JW, Hanson GR (2007) New insights into the mechanism of action of amphetamines. Annu Rev Pharmacol Toxicol 47: 681–698 10.1146/annurev.pharmtox.47.120505.105140 [doi]. 17209801 10.1146/annurev.pharmtox.47.120505.105140

[8] Sulzer D (2011) How addictive drugs disrupt presynaptic dopamine neurotransmission. Neuron 69: 628–649. 21338876 10.1016/j.neuron.2011.02.010 PMC3065181

[9] Paladini CA, Fiorillo CD, Morikawa H, Williams JT (2001) Amphetamine selectively blocks inhibitory glutamate transmission in dopamine neurons. Nat Neurosci 4: 275–281. 11224544 10.1038/85124

[10] Shi WX, Pun CL, Zhang XX, Jones MD, Bunney BS (2000) Dual effects of D-amphetamine on dopamine neurons mediated by dopamine and nondopamine receptors. J Neurosci 20: 3504–3511. 10777813 10.1523/JNEUROSCI.20-09-03504.2000 PMC6773133

[11] May LJ, Kuhr WG, Wightman RM (1988) Differentiation of dopamine overflow and uptake processes in the extracellular fluid of the rat caudate nucleus with fast-scan in vivo voltammetry. J Neurochem 51: 1060–1069. 2971098 10.1111/j.1471-4159.1988.tb03069.x

[12] Ramsson ES, Covey DP, Daberkow DP, Litherland MT, Juliano SA, et al (2011) Amphetamine augments action potential-dependent dopaminergic signaling in the striatum in vivo. J Neurochem 117: 937–948 10.1111/j.1471-4159.2011.07258.x [doi]. 21443523 10.1111/j.1471-4159.2011.07258.x PMC3134290

[13] Daberkow DP, Brown HD, Bunner KD, Kraniotis SA, Doellman MA, et al (2013) Amphetamine paradoxically augments exocytotic dopamine release and phasic dopamine signals. J Neurosci 33: 452–463 33/2/452 [pii];10.1523/JNEUROSCI.2136-12.2013 [doi]. 23303926 10.1523/JNEUROSCI.2136-12.2013 PMC3711765

[14] Ramsson ES, Howard CD, Covey DP, Garris PA (2011) High doses of amphetamine augment, rather than disrupt, exocytotic dopamine release in the dorsal and ventral striatum of the anesthetized rat. J Neurochem 119: 1162–1172 10.1111/j.1471-4159.2011.07407.x [doi]. 21806614 10.1111/j.1471-4159.2011.07407.x PMC3213283

[15] Schultz W (2007) Multiple dopamine functions at different time courses. Annu Rev Neurosci 30: 259–288 10.1146/annurev.neuro.28.061604.135722 [doi]. 17600522 10.1146/annurev.neuro.28.061604.135722

[16] Rizzoli SO, Betz WJ (2005) Synaptic vesicle pools. Nat Rev Neurosci 6: 57–69 nrn1583 [pii];10.1038/nrn1583 [doi]. 15611727 10.1038/nrn1583

[17] Ewing AG, Bigelow JC, Wightman RM (1983) Direct in vivo monitoring of dopamine released from two striatal compartments in the rat. Science 221: 169–171. 6857277 10.1126/science.6857277

[18] Michael AC, Ikeda M, Justice JB Jr (1987) Dynamics of the recovery of releasable dopamine following electrical stimulation of the medial forebrain bundle. Neurosci Lett 76: 81–86. 3495755 10.1016/0304-3940(87)90196-0

[19] Michael AC, Ikeda M, Justice JB Jr (1987) Mechanisms contributing to the recovery of striatal releasable dopamine following MFB stimulation. Brain Res 421: 325–335. 3500755 10.1016/0006-8993(87)91302-3

[20] Yavich L, MacDonald E (2000) Dopamine release from pharmacologically distinct storage pools in rat striatum following stimulation at frequency of neuronal bursting. Brain Res 870: 73–79. 10869503 10.1016/s0006-8993(00)02403-3

[21] Anderson BB, Chen G, Gutman DA, Ewing AG (1998) Dopamine levels of two classes of vesicles are differentially depleted by amphetamine. Brain Res 788: 294–301. 9555063 10.1016/s0006-8993(98)00040-7

[22] Chen G, Ewing AG (1995) Multiple classes of catecholamine vesicles observed during exocytosis from the Planorbis cell body. Brain Res 701: 167–174 0006-8993(95)00989-9 [pii]. 8925280 10.1016/0006-8993(95)00989-9

[23] Riddle EL, Hanson GR, Fleckenstein AE (2007) Therapeutic doses of amphetamine and methylphenidate selectively redistribute the vesicular monoamine transporter-2. Eur J Pharmacol 571: 25–28 S0014-2999(07)00645-0 [pii];10.1016/j.ejphar.2007.05.044 [doi]. 17618619 10.1016/j.ejphar.2007.05.044 PMC2581712

[24] Dugast C, Suaud-Chagny MF, Gonon F (1994) Continuous in vivo monitoring of evoked dopamine release in the rat nucleus accumbens by amperometry. Neuroscience 62: 647–654. 7870296 10.1016/0306-4522(94)90466-9

[25] Stamford JA, Kruk ZL, Millar J (1986) Measurement of stimulated dopamine release in the rat by in vivo voltammetry: the influence of stimulus duration on drug responses. Neurosci Lett 69: 70–73 0304-3940(86)90416-7 [pii]. 3748467 10.1016/0304-3940(86)90416-7

[26] Robinson DL, Hermans A, Seipel AT, Wightman RM (2008) Monitoring rapid chemical communication in the brain. Chem Rev 108: 2554–2584 10.1021/cr068081q [doi]. 18576692 10.1021/cr068081q PMC3110685

[27] Cheer JF, Heien ML, Garris PA, Carelli RM, Wightman RM (2005) Simultaneous dopamine and single-unit recordings reveal accumbens GABAergic responses: implications for intracranial self-stimulation. Proc Natl Acad Sci U S A 102: 19150–19155 0509607102 [pii];10.1073/pnas.0509607102 [doi]. 16380429 10.1073/pnas.0509607102 PMC1323210

[28] Wightman RM, Amatore C, Engstrom RC, Hale PD, Kristensen EW, et al (1988) Real-time characterization of dopamine overflow and uptake in the rat striatum. Neuroscience 25: 513–523. 3399057 10.1016/0306-4522(88)90255-2

[29] Wu Q, Reith ME, Wightman RM, Kawagoe KT, Garris PA (2001) Determination of release and uptake parameters from electrically evoked dopamine dynamics measured by real-time voltammetry. J Neurosci Methods 112: 119–133. 11716947 10.1016/s0165-0270(01)00459-9

[30] Paxinos G, Watson C (1986) The rat brain in stereotaxic coordinates. New York: Academic Press.

[31] Wu Q, Reith ME, Kuhar MJ, Carroll FI, Garris PA (2001) Preferential increases in nucleus accumbens dopamine after systemic cocaine administration are caused by unique characteristics of dopamine neurotransmission. J Neurosci 21: 6338–6347 21/16/6338 [pii]. 11487657 10.1523/JNEUROSCI.21-16-06338.2001 PMC6763153

[32] Michael D, Travis ER, Wightman RM (1998) Color images for fast-scan CV measurements in biological systems. Anal Chem 70: 586A–592A. 10.1021/ac9819640 9737201

[33] John CE, Jones SR (2007) Voltammetric characterization of the effect of monoamine uptake inhibitors and releasers on dopamine and serotonin uptake in mouse caudate-putamen and substantia nigra slices. Neuropharmacology 52: 1596–1605. 17459426 10.1016/j.neuropharm.2007.03.004 PMC2041899

[34] Keithley RB, Wightman RM (2011) Assessing principal component regression prediction of neurochemicals detected with fast-scan cyclic voltammetry. ACS Chem Neurosci 2: 514–525 10.1021/cn200035u [doi]. 21966586 10.1021/cn200035u PMC3182154

[35] Hermans A, Keithley RB, Kita JM, Sombers LA, Wightman RM (2008) Dopamine detection with fast-scan cyclic voltammetry used with analog background subtraction. Anal Chem 80: 4040–4048 10.1021/ac800108j [doi]. 18433146 10.1021/ac800108j

[36] Aragona BJ, Cleaveland NA, Stuber GD, Day JJ, Carelli RM, et al (2008) Preferential enhancement of dopamine transmission within the nucleus accumbens shell by cocaine is attributable to a direct increase in phasic dopamine release events. J Neurosci 28: 8821–8831 28/35/8821 [pii];10.1523/JNEUROSCI.2225-08.2008 [doi]. 18753384 10.1523/JNEUROSCI.2225-08.2008 PMC2584805

[37] Wightman RM, Heien ML, Wassum KM, Sombers LA, Aragona BJ, et al (2007) Dopamine release is heterogeneous within microenvironments of the rat nucleus accumbens. Eur J Neurosci 26: 2046–2054 EJN5772 [pii];10.1111/j.1460-9568.2007.05772.x [doi]. 17868375 10.1111/j.1460-9568.2007.05772.x

[38] Koulchitsky S, De BB, Quertemont E, Charlier C, Seutin V (2012) Differential effects of cocaine on dopamine neuron firing in awake and anesthetized rats. Neuropsychopharmacology 37: 1559–1571 npp2011339 [pii];10.1038/npp.2011.339 [doi]. 22298123 10.1038/npp.2011.339 PMC3358732

[39] Kuhr WG, Wightman RM, Rebec GV (1987) Dopaminergic neurons: simultaneous measurements of dopamine release and single-unit activity during stimulation of the medial forebrain bundle. Brain Res 418: 122–128 0006-8993(87)90968-1 [pii]. 3499205 10.1016/0006-8993(87)90968-1

[40] Montague PR, McClure SM, Baldwin PR, Phillips PE, Budygin EA, et al (2004) Dynamic gain control of dopamine delivery in freely moving animals. J Neurosci 24: 1754–1759 10.1523/JNEUROSCI.4279-03.2004 [doi];24/7/1754 [pii]. 14973252 10.1523/JNEUROSCI.4279-03.2004 PMC6730459

[41] Venton BJ, Zhang H, Garris PA, Phillips PE, Sulzer D, et al (2003) Real-time decoding of dopamine concentration changes in the caudate-putamen during tonic and phasic firing. J Neurochem 87: 1284–1295 2109 [pii]. 14622108 10.1046/j.1471-4159.2003.02109.x

[42] Borland LM, Shi G, Yang H, Michael AC (2005) Voltammetric study of extracellular dopamine near microdialysis probes acutely implanted in the striatum of the anesthetized rat. J Neurosci Methods 146: 149–158 S0165-0270(05)00048-8 [pii];10.1016/j.jneumeth.2005.02.002 [doi]. 15975664 10.1016/j.jneumeth.2005.02.002

[43] Kelland MD, Chiodo LA, Freeman AS (1990) Anesthetic influences on the basal activity and pharmacological responsiveness of nigrostriatal dopamine neurons. Synapse 6: 207–209 10.1002/syn.890060213 [doi]. 1978421 10.1002/syn.890060213

[44] Owesson-White CA, Roitman MF, Sombers LA, Belle AM, Keithley RB, et al (2012) Sources contributing to the average extracellular concentration of dopamine in the nucleus accumbens. J Neurochem 121: 252–262 10.1111/j.1471-4159.2012.07677.x [doi]. 22296263 10.1111/j.1471-4159.2012.07677.x PMC3323736

[45] Oleson EB, Salek J, Bonin KD, Jones SR, Budygin EA (2009) Real-time voltammetric detection of cocaine-induced dopamine changes in the striatum of freely moving mice. Neurosci Lett 467: 144–146. 19822192 10.1016/j.neulet.2009.10.025 PMC2787452

[46] Stuber GD, Roitman MF, Phillips PE, Carelli RM, Wightman RM (2005) Rapid dopamine signaling in the nucleus accumbens during contingent and noncontingent cocaine administration. Neuropsychopharmacology 30: 853–863. 15549053 10.1038/sj.npp.1300619

[47] Kuhr WG, Ewing AG, Near JA, Wightman RM (1985) Amphetamine attenuates the stimulated release of dopamine in vivo. J Pharmacol Exp Ther 232: 388–394. 3968641

[48] Kuhr WG, Bigelow JC, Wightman RM (1986) In vivo comparison of the regulation of releasable dopamine in the caudate nucleus and the nucleus accumbens of the rat brain. J Neurosci 6: 974–982. 3486259 10.1523/JNEUROSCI.06-04-00974.1986 PMC6568443

[49] Jones SR, Garris PA, Wightman RM (1995) Different effects of cocaine and nomifensine on dopamine uptake in the caudate-putamen and nucleus accumbens. J Pharmacol Exp Ther 274: 396–403. 7616424

[50] Scorza MC, Carrau C, Silveira R, Zapata-Torres G, Cassels BK, et al (1997) Monoamine oxidase inhibitory properties of some methoxylated and alkylthio amphetamine derivatives: structure-activity relationships. Biochem Pharmacol 54: 1361–1369 S0006-2952(97)00405-X [pii]. 9393679 10.1016/s0006-2952(97)00405-x

[51] Kuczenski R (1975) Effects of catecholamine releasing agents on synaptosomal dopamine biosynthesis: multiple pools of dopamine or multiple forms of tyrosine hydroxylase. Neuropharmacology 14: 1–10. 239360 10.1016/0028-3908(75)90060-x

[52] Ingram SL, Prasad BM, Amara SG (2002) Dopamine transporter-mediated conductances increase excitability of midbrain dopamine neurons. Nat Neurosci 5: 971–978 10.1038/nn920 [doi];nn920 [pii]. 12352983 10.1038/nn920

[53] Mundorf ML, Hochstetler SE, Wightman RM (1999) Amine weak bases disrupt vesicular storage and promote exocytosis in chromaffin cells. J Neurochem 73: 2397–2405. 10582599 10.1046/j.1471-4159.1999.0732397.x

[54] Fleckenstein AE, Volz TJ, Hanson GR (2009) Psychostimulant-induced alterations in vesicular monoamine transporter-2 function: neurotoxic and therapeutic implications. Neuropharmacology 56 Suppl 1: 133–138 S0028-3908(08)00273-6 [pii];10.1016/j.neuropharm.2008.07.002 [doi]. 18662707 10.1016/j.neuropharm.2008.07.002 PMC2634813

[55] Volz TJ, Farnsworth SJ, King JL, Riddle EL, Hanson GR, et al (2007) Methylphenidate administration alters vesicular monoamine transporter-2 function in cytoplasmic and membrane-associated vesicles. J Pharmacol Exp Ther 323: 738–745 jpet.107.126888 [pii];10.1124/jpet.107.126888 [doi]. 17693585 10.1124/jpet.107.126888

[56] Bowyer JF, Masserano JM, Weiner N (1987) Inhibitory effects of amphetamine on potassium-stimulated release of [3H]dopamine from striatal slices and synaptosomes. J Pharmacol Exp Ther 240: 177–186. 3100768

[57] Floor E, Meng L (1996) Amphetamine releases dopamine from synaptic vesicles by dual mechanisms. Neurosci Lett 215: 53–56 S0304-3940(96)12963-3 [pii]. 8880752 10.1016/s0304-3940(96)12963-3

[58] Jones SR, Gainetdinov RR, Wightman RM, Caron MG (1998) Mechanisms of amphetamine action revealed in mice lacking the dopamine transporter. J Neurosci 18: 1979–1986. 9482784 10.1523/JNEUROSCI.18-06-01979.1998 PMC6792915

[59] Mosharov EV, Gong LW, Khanna B, Sulzer D, Lindau M (2003) Intracellular patch electrochemistry: regulation of cytosolic catecholamines in chromaffin cells. J Neurosci 23: 5835–5845 23/13/5835 [pii]. 12843288 10.1523/JNEUROSCI.23-13-05835.2003 PMC6741260

[60] Patel J, Mooslehner KA, Chan PM, Emson PC, Stamford JA (2003) Presynaptic control of striatal dopamine neurotransmission in adult vesicular monoamine transporter 2 (VMAT2) mutant mice. J Neurochem 85: 898–910 1732 [pii]. 12716422 10.1046/j.1471-4159.2003.01732.x

[61] Schmitz Y, Lee CJ, Schmauss C, Gonon F, Sulzer D (2001) Amphetamine distorts stimulation-dependent dopamine overflow: effects on D2 autoreceptors, transporters, and synaptic vesicle stores. J Neurosci 21: 5916–5924. 11487614 10.1523/JNEUROSCI.21-16-05916.2001 PMC6763160

[62] Cass WA, Gerhardt GA, Mayfield RD, Curella P, Zahniser NR (1992) Differences in dopamine clearance and diffusion in rat striatum and nucleus accumbens following systemic cocaine administration. J Neurochem 59: 259–266. 1613502 10.1111/j.1471-4159.1992.tb08899.x

[63] Marshall JF, O'Dell SJ, Navarrete R, Rosenstein AJ (1990) Dopamine high-affinity transport site topography in rat brain: major differences between dorsal and ventral striatum. Neuroscience 37: 11–21 0306-4522(90)90187-9 [pii]. 2243588 10.1016/0306-4522(90)90187-9

[64] Lew R, Patel A, Vaughan RA, Wilson A, Kuhar MJ (1992) Microheterogeneity of dopamine transporters in rat striatum and nucleus accumbens. Brain Res 584: 266–271 0006-8993(92)90905-O [pii]. 1515945 10.1016/0006-8993(92)90905-o

[65] Avelar AJ, Juliano SA, Garris PA (2013) Amphetamine augments vesicular dopamine release in the dorsal and ventral striatum through different mechanisms. J Neurochem in press. 10.1111/jnc.12197 PMC3633730 23406303

[66] Hondebrink L, Meulenbelt J, Timmerman JG, van den Berg M, Westerink RH (2009) Amphetamine reduces vesicular dopamine content in dexamethasone-differentiated PC12 cells only following L-DOPA exposure. J Neurochem 111: 624–633 JNC6357 [pii];10.1111/j.1471-4159.2009.06357.x [doi]. 19702656 10.1111/j.1471-4159.2009.06357.x

[67] Sitte HH, Huck S, Reither H, Boehm S, Singer EA, et al (1998) Carrier-mediated release, transport rates, and charge transfer induced by amphetamine, tyramine, and dopamine in mammalian cells transfected with the human dopamine transporter. J Neurochem 71: 1289–1297. 9721755 10.1046/j.1471-4159.1998.71031289.x

[68] Sulzer D, Chen TK, Lau YY, Kristensen H, Rayport S, et al (1995) Amphetamine redistributes dopamine from synaptic vesicles to the cytosol and promotes reverse transport. J Neurosci 15: 4102–4108. 7751968 10.1523/JNEUROSCI.15-05-04102.1995 PMC6578196

[69] Kile BM, Guillot TS, Venton BJ, Wetsel WC, Augustine GJ, et al (2010) Synapsins differentially control dopamine and serotonin release. J Neurosci 30: 9762–9770 30/29/9762 [pii];10.1523/JNEUROSCI.2071-09.2010 [doi]. 20660258 10.1523/JNEUROSCI.2071-09.2010 PMC2923550

[70] Lee TH, Balu R, Davidson C, Ellinwood EH (2001) Differential time-course profiles of dopamine release and uptake changes induced by three dopamine uptake inhibitors. Synapse 41: 301–310 10.1002/syn.1087 [pii];10.1002/syn.1087 [doi]. 11494401 10.1002/syn.1087

[71] Venton BJ, Seipel AT, Phillips PE, Wetsel WC, Gitler D, et al (2006) Cocaine increases dopamine release by mobilization of a synapsin-dependent reserve pool. J Neurosci 26: 3206–3209. 16554471 10.1523/JNEUROSCI.4901-04.2006 PMC6674099

[72] Benwell ME, Balfour DJ, Lucchi HM (1993) Influence of tetrodotoxin and calcium on changes in extracellular dopamine levels evoked by systemic nicotine. Psychopharmacology (Berl) 112: 467–474. 7871059 10.1007/BF02244896

[73] Nomikos GG, Damsma G, Wenkstern D, Fibiger HC (1990) In vivo characterization of locally applied dopamine uptake inhibitors by striatal microdialysis. Synapse 6: 106–112 10.1002/syn.890060113 [doi]. 1697988 10.1002/syn.890060113

[74] Westerink BH, Tuntler J, Damsma G, Rollema H, de Vries JB (1987) The use of tetrodotoxin for the characterization of drug-enhanced dopamine release in conscious rats studied by brain dialysis. Naunyn Schmiedebergs Arch Pharmacol 336: 502–507. 3501841 10.1007/BF00169306

[75] Cheer JF, Wassum KM, Sombers LA, Heien ML, Ariansen JL, et al (2007) Phasic dopamine release evoked by abused substances requires cannabinoid receptor activation. J Neurosci 27: 791–795 27/4/791 [pii];10.1523/JNEUROSCI.4152-06.2007 [doi]. 17251418 10.1523/JNEUROSCI.4152-06.2007 PMC6672925

[76] Robinson DL, Howard EC, McConnell S, Gonzales RA, Wightman RM (2009) Disparity between tonic and phasic ethanol-induced dopamine increases in the nucleus accumbens of rats. Alcohol Clin Exp Res 33: 1187–1196 ACER942 [pii];10.1111/j.1530-0277.2009.00942.x [doi]. 19389195 10.1111/j.1530-0277.2009.00942.x PMC2947861

[77] Hyman SE, Malenka RC, Nestler EJ (2006) Neural mechanisms of addiction: the role of reward-related learning and memory. Annu Rev Neurosci 29: 565–598 10.1146/annurev.neuro.29.051605.113009 [doi]. 16776597 10.1146/annurev.neuro.29.051605.113009

[78] Wiecki TV, Frank MJ (2010) Neurocomputational models of motor and cognitive deficits in Parkinson's disease. Prog Brain Res 183: 275–297 S0079-6123(10)83014-6 [pii];10.1016/S0079-6123(10)83014-6 [doi]. 20696325 10.1016/S0079-6123(10)83014-6

[79] Everitt BJ, Robbins TW (2005) Neural systems of reinforcement for drug addiction: from actions to habits to compulsion. Nat Neurosci 8: 1481–1489 nn1579 [pii];10.1038/nn1579 [doi]. 16251991 10.1038/nn1579

[80] Volkow ND, Wang GJ, Fowler JS, Tomasi D, Telang F (2011) Addiction: beyond dopamine reward circuitry. Proc Natl Acad Sci U S A 108: 15037–15042 1010654108 [pii];10.1073/pnas.1010654108 [doi]. 21402948 10.1073/pnas.1010654108 PMC3174598

